# Anti-demineralizing protective effects on enamel identified in experimental and commercial restorative materials with functional fillers

**DOI:** 10.1038/s41598-021-91279-z

**Published:** 2021-06-03

**Authors:** Matej Par, Andrea Gubler, Thomas Attin, Zrinka Tarle, Tobias T. Tauböck

**Affiliations:** 1 Department of Endodontics and Restorative Dentistry, School of Dental Medicine, University of Zagreb, Gunduliceva 5, Zagreb, Croatia; 2 Department of Conservative and Preventive Dentistry, Center for Dental Medicine, University of Zurich, Plattenstrasse 11, Zurich, Switzerland

**Keywords:** Composite resin, Dental biomaterials

## Abstract

The aim of this study was to investigate whether experimental and commercial dental restorative materials with functional fillers can exert a protective anti-demineralizing effect on enamel that is not immediately adjacent to the restoration. Four experimental resin composites with bioactive glass and three commercial restorative materials were investigated. Enamel blocks were incubated in a lactic acid solution (pH = 4.0) at a standardized distance (5 mm) from cured specimens of restorative materials. The lactic acid solution was replenished every 4 days up to a total of 32 days. Surfaces of enamel blocks were periodically evaluated by Knoop microhardness measurements and scanning electron microscopy. The protective effect of restorative materials against acid was identified as enamel microhardness remaining unchanged for a certain number of 4-day acid addition cycles. Additionally, the pH of the immersion medium was measured. While enamel microhardness in the control group was maintained for 1 acid addition cycle (4 days), restorative materials postponed enamel softening for 2–5 cycles (8–20 days). The materials capable of exerting a stronger alkalizing effect provided longer-lasting enamel protection. The protective and alkalizing effects of experimental composites improved with higher amounts of bioactive glass and were better for conventional bioactive glass 45S5 compared to a fluoride-containing bioactive glass. Scanning electron micrographs evidenced the protective effect of restorative materials by showing a delayed appearance of an etching pattern on the enamel surface. A remotely-acting anti-demineralizing protective effect on enamel was identified in experimental composites functionalized with two types of bioactive glass, as well as in three commercial ion-releasing restorative materials.

## Introduction

As secondary caries represents one of the main reasons for the failure of composite restorations^1^, bioactive glasses (BGs) have been investigated as interesting dopants for restorative resin composites due to their potential for releasing remineralizing ions^2, 3^, neutralizing acid^4, 5^, and precipitating hydroxyapatite^6, 7^. BGs encompass various formulations with adjustable relative ratios of constituent elements. The composition of a particular BG determines its network structure, which in turn reflects on its properties^8^. In this way, subtle compositional adjustments can be made to tailor reactivity, solubility, and ion release for a desired therapeutic effect^9^. Among various BG compositions that have been investigated as functional additives for methacrylate-based restorative composites, fluoride-modified BGs appear especially promising due to their ability for releasing fluoride ions when exposed to an aqueous medium^2^. These ions can be incorporated into the enamel to render it more resistant to demineralization^10^. An additional potential benefit of fluoride-modified BGs is their capability to form a fluorapatite layer on the composite surface^11^, which can be envisioned as a possible sealing agent for marginal gaps^12^. Besides experimental BG formulations containing various therapeutic elements, the conventional Hench’s BG 45S5 formulation has also been extensively investigated as a possible filler for resin composites^3–6, 13^. Notwithstanding the unavoidable trade-offs between its high reactivity and stability of mechanical properties^14^ and the lack of fluoride release, the traditional BG 45S5 composition represents a promising candidate for functionalizing resin composites^4, 6, 15, 16^.

BG embedded within resin composites dissolves when exposed to an aqueous medium, releasing its constituent elements into the solution^2^. This ion release is accompanied by an alkalizing effect^11^. The release of remineralizing ions and the increase of the solution pH can be employed to protect dental hard tissues against demineralization caused by bacterial acids. Such a protective effect has been demonstrated in experimental BG-functionalized composites intended for use as orthodontic adhesives; these materials showed the potential for protecting enamel adjacent to bonded brackets against the formation of white spots^17–21^. To investigate whether the protective effect of BG-functionalized composites can extend beyond the dental hard tissues immediately adjacent to the material^22^, the present in vitro study used enamel blocks incubated in a lactic acid solution at a standardized distance from cured specimens of restorative materials. Experimental composites functionalized with 10 or 20 wt% of conventional BG 45S5 and an experimental fluoride-modified BG were compared to three commercial restorative materials capable of exerting an anti-demineralizing protective effect. Microhardness (MH) was chosen as a convenient indicator of structural integrity of the enamel surface^23^ exposed to simulated cycles of acid attack. Additionally, enamel surface morphology and pH changes in the immersion solution were evaluated. The null hypotheses were that the evaluated parameters (enamel MH, enamel surface morphology, and pH of the immersion solution) would: (1) remain unchanged during the simulated acid attack; and (2) not differ among the investigated restorative materials.

## Materials and methods

### Experimental resin composites

Experimental resin composites were prepared as described in previous studies^11, 24^. The resin system contained bisphenol-A-glycidyldimethacrylate (Bis-GMA, Merck, Darmstadt, Germany) and triethylene glycol dimethacrylate (TEGDMA, Merck) in a ratio of 60:40 wt%. Camphorquinone (0.2 wt%; Merck) and ethyl-4-(dimethylamino) benzoate (0.8 wt%; Merck) were added to photoactivate the resin system. The components of the resin system were mixed using a magnetic stirrer for 48 h.

The composition of fillers used to prepare experimental composites is given in Table 1. BG 45S5, inert barium glass, and silica were obtained from commercial vendors. The experimental fluoride-modified experimental BG with theoretical network connectivity similar to that of BG 45S5 (2.1)^8^ was prepared on-demand by Schott (Mainz, Germany) via melt-quench route. By using similar preparation and grinding procedures, comparable particle sizes were obtained for both BG types.

**Table 1:** Bioactive glass and reinforcing fillers used in experimental composites.

	Bioactive glass 45S5	Experimental fluoride-containing bioactive glass	Inert barium glass	Silica
Particle size (d50)	3 µm	3 µm	1 µm	5–50 nm
Composition (wt%)	45.0% SiO_2_ 24.5% CaO 24.5% Na_2_O 6.0% P_2_O_5_	33.5% SiO_2_ 33.0% CaO 10.5% Na_2_O 11.0% P_2_O_5_ 12.0% CaF_2_	55.0% SiO_2_ 25.0% BaO 10.0% Al_2_O_3_ 10.0% B_2_O_3_	> 99.8% SiO_2_
Silanization (wt%)	None	None	3.2	4–6
Manufacturer	Schott, Mainz, Germany	Schott, Mainz, Germany	Schott, Mainz, Germany	Evonik, Hanau, Germany
Product name / LOT	G018-144 / M111473	Experimental batch	GM27884 / Sil13696	Aerosil R 7200 / 157,020,635

Experimental composites with a total filler ratio of 70 wt% were prepared by replacing 0, 10, or 20 wt% of silanized reinforcing fillers (barium glass and silica) with two types of unsilanized BG (Table 2). The experimental composite containing only reinforcing fillers was used as an inert control. The composites were prepared by mixing the resin system and the fillers for 5 min in a dual asymmetric centrifugal mixing system (Speed Mixer TM DAC 150 FVZ, Hauschild & Co. KG, Hamm, Germany) at 2,000 rpm, and deaerating in vacuum for 48 h.

**Table 2:** Composition of experimental composites.

Material designation	Filler composition (wt%)			Total filler ratio (wt%)
	Bioactive glass 45S5	Experimental fluoride-containing bioactive glass	Reinforcing fillers (inert barium glass : silica = 2:1)	
Control	0	0	70	70
C-10	10	0	60	70
C-20	20	0	50	70
E-10	0	10	60	70
E-20	0	20	50	70

In addition to the experimental composites, three commercial restorative materials with an acid-protective capability were used as references: a reinforced glass ionomer restorative (ChemFil Rock, Dentsply Sirona, Konstanz, Germany; shade: A2, LOT: 1903000819), a giomer (Beautifil II, Shofu, Kyoto, Japan; shade: A2, LOT: 041923), and a resin-based “alkasite” material (Cention, Ivoclar Vivadent, Schaan, Liechtenstein; shade: universal, LOT: XL7102). The alkasite material contains two types of reactive filers: an ionomer glass based on a calcium barium alumino-fluoro-silicate, and a calcium fluoro-silicate glass^25, 26^.

### Enamel blocks

One hundred twenty-eight intact human third molars were collected as by-products of regular dental treatment. Patients had given written informed consent to use the teeth for research purposes, and all teeth were irreversibly anonymized immediately after extraction. Under these terms, the research complied with the use of anonymized biological material and, consequently, authorization from the local ethics committee was not required (Federal Act on Research involving Human Beings (Human Research Act; article 2, paragraph 2)). The teeth were stored at 8 °C in 0.1% thymol solution and used within 6 months of extraction.

**Figure 1:**
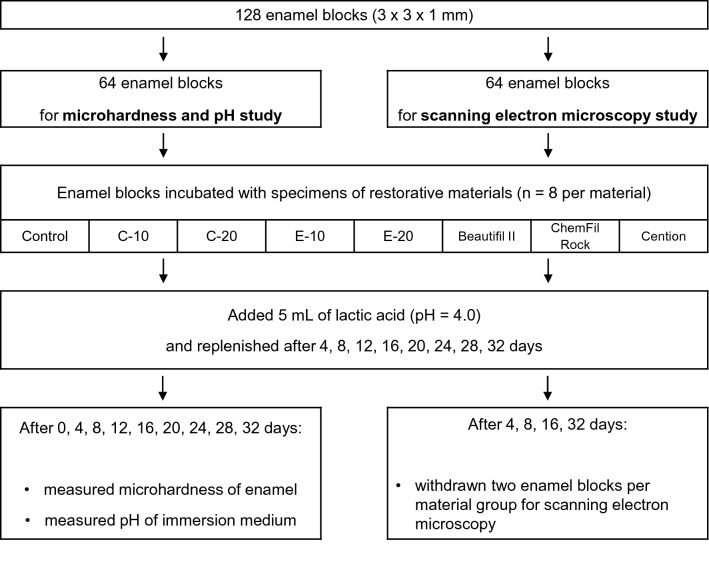
Experimental design.

Enamel blocks (1 block per tooth; 3 × 3 × 1 mm) were prepared from buccal surfaces using a low-speed precision cutting machine (IsoMet, Buehler, Lake Bluff, IL, USA). The buccal sides of enamel blocks were ground using P4000 silicon carbide paper^27^ (Buehler; 2 min at 30 rpm, median particle size = 2.5 µm). After preparation, the enamel blocks were stored in a phosphate-buffered saline solution and used within five days of preparation. From the total of 128 enamel blocks, 64 were used for the MH and pH measurements, while 64 were used for the scanning electron microscopy (SEM) study, as described in the schematic representation of the study design in Fig. 1.

### Restorative material specimens

Discoid specimens (diameter = 7 mm, thickness = 2 mm) were prepared by casting the restorative materials into custom-made polyoxymethylene molds, covering their surfaces with Mylar foils, and flattening using glass plates. The glass ionomer material was left to set in the mold for 15 min, while the light-curable materials (giomer and experimental composites) and the dual-curable alkasite material were illuminated using a violet-blue LED curing unit (Bluephase PowerCure, Ivoclar Vivadent, Schaan, Liechtenstein; emission wavelength range: 390–500 nm, radiant exitance: 1,340 mW/cm^2^) for 20 s, resulting in a total radiant exposure of 26.8 J/cm^2^). To simulate a clinically realistic scenario, the specimens were exposed to the immersion medium within 15 min after preparation. Per material, 16 specimens were prepared (8 for the MH and pH study; and 8 for the SEM study).

### Immersion in lactic acid

The enamel blocks and restorative material specimens were immersed in closed vials (Eppendorf; Hamburg, Germany) containing 5 mL of lactic acid solution (pH = 4.0). This experimental setup was adopted and modified according to previous studies^28–30^. Each vial contained one enamel block and one restorative material specimen, which were held at a standardized distance of 5 mm. The immersion solution was agitated using a horizontal laboratory shaker at a speed of 30 revolutions per minute. The environmental temperature ranged between 23–24 °C. Surface MH of enamel blocks and pH of the immersion solution were measured at the following time points: 4, 8, 12, 16, 20, 24, 28, and 32 days. At each time point, the immersion medium was replenished with 5 mL of fresh lactic acid solution (pH = 4.0).

### Microhardness measurements

Knoop MH measurements were performed on the buccal sides of enamel blocks using a digital hardness tester (model no. 1600–6106; Buehler). Indentations were made under a load of 100 g and a dwell time of 20 s at random positions. The indentations were evaluated within 2 min after preparation, with a resolution of 0.015 µm^31^. Per each specimen and time point, five replicate indentations were made and their mean values were considered as a statistical unit. Eight specimens were used per material (n = 8).

### pH measurements

A calibrated pH electrode (780 pH Meter, Metrohm, Herisau, Switzerland) was immersed in the solution, and pH values were recorded with a resolution of 0.01 pH units. Per each specimen and time point, three replicate measurements were made and their mean values were considered as a statistical unit. Eight specimens were used per material (n = 8).

### Scanning electron microscopy

Enamel blocks for the SEM study (n = 8 per material) were subjected to the same acid exposure protocol as the specimens for the evaluation of MH and pH. Upon reaching the time points of 4, 8, 16, and 32 days, two enamel blocks per material were withdrawn, rinsed with distilled water, dried, and sputtered with 5 nm of gold. A scanning electron microscope (SEM; GeminiSEM 450, Zeiss, Oberkochen, Germany) was used at 10 kV and 10,000 × magnification to evaluate surface morphology of enamel blocks.

### Statistical analysis

Normality of distribution and homogeneity of variances were checked using Levene’s and Shapiro–Wilk’s tests, respectively. Within each restorative material, MH and pH values were compared among time points using repeated-measures ANOVA with Bonferroni correction for multiple comparisons. The statistical analysis was performed using SPSS (version 20, IBM, Armonk, NY, USA) at an overall level of significance of α = 0.05.

## Results

**Figure 2:**
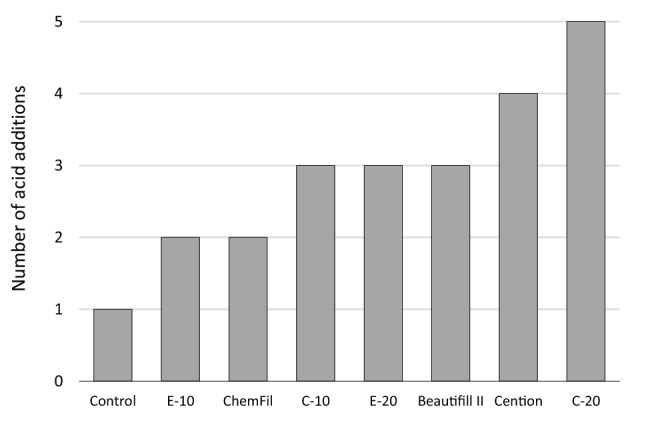
Number of acid addition cycles for which enamel surface MH remained statistically similar to initial values.

MH values of enamel blocks measured after acid addition cycles are shown in Table 3. Repeated additions of acid solution significantly decreased enamel MH in all groups. The protective effect of restorative materials against acid was identified as enamel MH remaining statistically similar to initial MH values for a certain number of acid additions. The enamel MH in the control group was maintained for up to 1 acid addition and showed a statistically significant decrease thereafter. In all other groups, enamel MH was maintained for more acid addition cycles, remaining statistically similar to initial MH values for up to 2 cycles (E-10 and ChemFil), 3 cycles (C-10, E-20, and Beautifil II), 4 cycles (Cention) and 5 cycles (C-20). The number of acid additions over which enamel MH remained statistically similar to the initial values is summarized in Fig. 2.

**Table 3:** Knoop microhardness of enamel blocks (mean values with standard deviations in parentheses).

Time	No. of acid addition cycles	Material							
		Control	C-10	C-20	E-10	E-20	Beautifil II	ChemFil	Cention
Initial	0	243.8 (38.8) a	243.7 (39.2) ab	255.2 (42.9) a	262.4 (20.5) a	233.8 (45.0) a	252.7 (33.9) a	267.6 (22.2) a	261.9 (28.6) ab
4 days	1	228.3 (29.8) a	254.2 (32.6) a	267.6 (38.1) a	259.3 (25.1) a	263.8 (21.5) a	245.9 (27.2) a	260.5 (22.2) ab	276.4 (29.8) a
8 days	2	174.9 (32.3) b	207.2 (27.3) bc	256.6 (33.9) a	231.1 (34.1) a	245.4 (40.3) a	203.1 (33.1) ab	244.8 (23.8) abc	269.9 (20.5) a
12 days	3	145.8 (26.8) bc	178.5 (29.8) bc	239.7 (40.4) a	220.6 (22.1) b	221.0 (35.1) ab	201.6 (42.7) ab	221.4 (32.7) bc	239.3 (27.8) ab
16 days	4	107.1 (33.0) cd	136.1 (23.4) d	230.2 (51.8) a	167.2 (32.5) c	174.9 (37.5) bc	175.3 (33.1) b	203.5 (39.5) bc	212.8 (28.2) bc
20 days	5	81.8 (13.7) de	113.9 (32.6) d	207.7 (49.7) ab	123.5 (32.4) d	136.1 (40.0) c	156.9 (36.8) bc	198.5 (47.6) c	175.0 (50.1) c
24 days	6	40.7 (20.3) ef	47.1 (11.6) e	147.4 (51.6) bc	55.5 (30.7) e	69.4 (27.9) d	99.3 (36.5) d	129.4 (44.0) d	98.2 (50.3) d
28 days	7	29.4 (10.6) f	38.6 (14.8) e	145.1 (48.1) bc	43.0 (23.2) e	59.2 (19.1) d	106.0 (29.1) cd	112.4 (49.6) d	70.5 (30.6) d
32 days	8	25.3 (5.3) f	30.8 (9.2) e	122.3 (41.5) c	36.0 (11.4) e	50.0 (16.0) d	88.9 (35.3) d	113.4 (48.6) d	63.8 (19.5) d

Same letters denote statistically similar microhardness values within a material.

**Figure 3:**
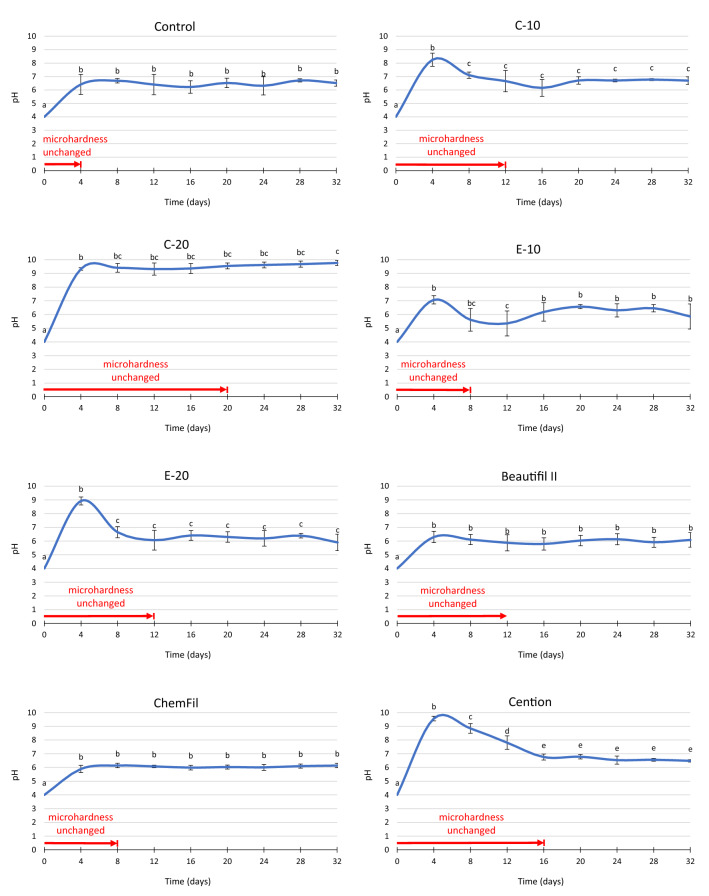
pH changes (mean values ± standard deviations) of the immersion medium and time periods for which enamel microhardness remained unchanged compared to initial values. Same letters denote statistically similar pH values within a material.

The pH changes in the immersion solution are shown in Fig. 3. The initial pH (measured before incubating enamel blocks and restorative materials) was 4.0 in all groups. Statistically significant pH changes were observed after periodic acid additions, leading to the following three patterns in the pH curves:

1. A significant initial pH increase, followed by a plateau at pH = 6–7. Materials: Control, E-10, Beautifil II, and ChemFil.
2. A transient peak at pH = 8–10 after 4 days, followed by a pH drop and stabilization at pH = 6–7. Materials: C-10, E-20, and Cention.
3. A significant initial pH increase, followed by a plateau at pH = 9–10. Material: C-20.

The pH changes in patterns (1) and (2) converged to a plateau at pH = 6–7; this was the most common behavior identified in 7 out of 8 tested materials. Pattern (3) was observed for only one material (C-20) capable of maintaining alkaline pH over the whole observation period.

**Figure 4:**
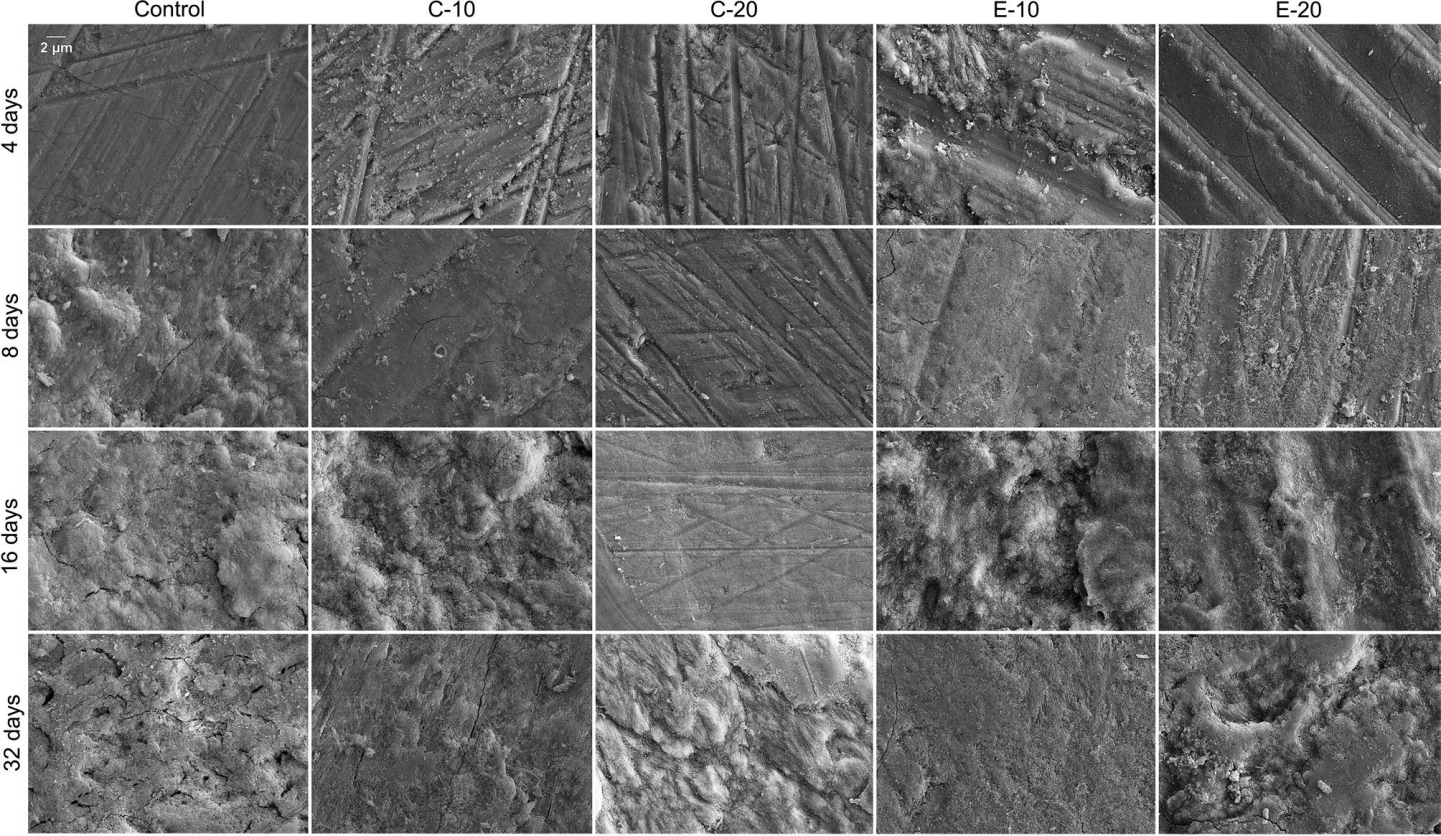
Scanning electron microscopy images of the surfaces of enamel blocks immersed with the control composite and the composites containing conventional (C-10 and C-20) and experimental (E-10 and E-20) bioactive glass.

**Figure 5:**
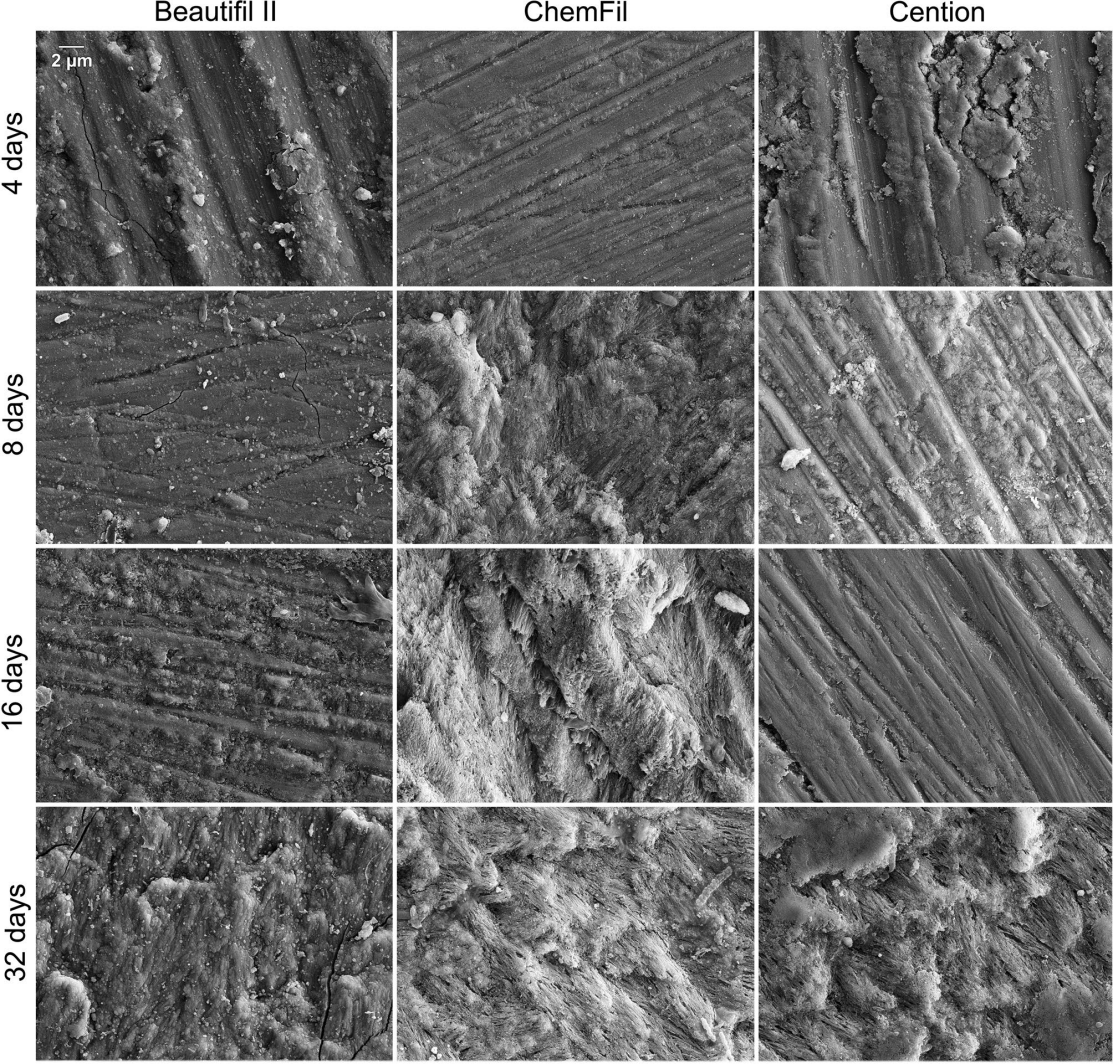
Scanning electron microscopy images of the surfaces of enamel blocks immersed with commercial reference materials.

SEM images of enamel surfaces after 4, 8, 16, and 32 days of immersion in the acid solution are shown in Fig. 4 for the experimental materials and Fig. 5 for the commercial materials. After 4 days, enamel surfaces in all groups showed a scratch pattern from grinding, while enamel blocks immersed with Cention additionally showed a precipitate on their surface. Over the subsequent time points (8, 16, and 32 days), the scratch patterns gradually became less visible and were replaced with etching patterns. For C-20, Beautifil II, and Cention, the scratch pattern remained visible up to 16 days, whereas for other materials they were replaced by etching patterns at earlier times (4–8 days). After 32 days, etching patterns were clearly observable for all materials.

## Discussion

This study showed that restorative materials with functional fillers exert an anti-demineralizing effect when incubated with enamel blocks in an acid solution. This effect was evidenced by MH of enamel blocks remaining unimpaired over a material-dependent number of acid attack cycles. To demonstrate that the protective effect does not depend on the close proximity of restorative materials to dental hard tissues, enamel blocks and restorative material specimens were separated by a distance of 5 mm. The protective effect was material-dependent and identified for the experimental BG-containing composites, as well as for three commercial restorative materials, leading us to reject both null hypotheses.

The composition of experimental composites with 10 and 20 wt% of BG followed a previous study in which these materials were shown to neutralize acid similarly to the alkasite material and better than a giomer and a glass ionomer^11^. These results motivated the present study, which aimed to investigate whether the acid-neutralizing and ion-releasing activities of experimental BG-composites can protect remote enamel surfaces against demineralization caused by lactic acid.

Notwithstanding the complex pathophysiology of tooth caries, the initial damage to the enamel surface stems from an imbalance in the dissolution and precipitation processes^32^. While various sophisticated approaches are beneficial for in-depth evaluations of caries progression^23^, simple measurements of enamel surface MH indicating initial structural changes due to acid exposure have proven useful for evaluations of the anti-demineralizing effect of restorative materials^33–35^. Therefore, our study employed periodic replenishing of the lactic acid solution and evaluated the number of acid addition cycles that enamel blocks withstood before showing signs of surface softening. The initial enamel MH in all experimental groups was within the usual MH range for sound enamel^36^. Statistical comparisons of those initial MH values and MH measured after successive acid additions enabled identifying the point of significant MH decrease, which was considered to signify initial enamel softening.

The capability of the investigated materials for protecting enamel against demineralization is based on two main effects, namely the neutralization of acid and the release of remineralizing ions^2, 11^. The latter mechanism shifts the dissolution/precipitation dynamics between the enamel hydroxyapatite and the immersion solution towards precipitation. Sufficiently saturating the immersion solution with calcium and phosphate ions can prevent enamel demineralization even in moderately acidic environments^32^. A strong alkalizing effect is therefore not necessary for a protective effect if sufficient concentrations of remineralizing ions are released. This consideration can explain the finding that E-10, Beautifil II, and ChemFil demonstrated a better protective effect than the control composite by maintaining enamel MH unchanged for a longer time (8–12 days vs. 4 days for the control composite) despite showing pH curves similar to that of the control composite. A comparatively better protective effect (MH unchanged for 12–20 days) was identified for the materials with a pronounced alkalizing capability, namely C-10, C-20, E-20, and Cention. For these materials, the synergistic effect of the alkalization and ion release led to a longer-lasting protective capability.

The pH increase observed for the control group can be attributed to the dissolution of enamel^37^ which led to acid neutralization, as previously reported in studies that incubated enamel blocks in lactic acid solution^28, 30^. As the control group showed a plateau at the pH = 6–7 throughout the whole observation period, the alkalizing effect in other groups had to be sufficiently pronounced in order to become distinguishable from the acid neutralization by enamel blocks alone. Such an effect was observed for C-10, E-20, and Cention showing a transient alkalization, and for C-20 which showed a more notable alkalization by maintaining a plateau at pH = 9–10. The observation of the alkalizing effect of E-10, Beautifil II, and ChemFil being similar to that of the control composite does not necessarily imply that these materials lack an acid-neutralizing effect. These materials indeed demonstrated a moderate acid-neutralizing effect in a previous study^11^; however, by measuring pH at the end of four-day acid addition cycles in the present study, their contributions to the pH increase were indiscernible from the acid-neutralizing effect of the dissolution of enamel blocks.

As the acid-neutralizing effect was eventually exhausted for all materials except C-20, their pH curves converged to the plateau values of the control composite at pH = 6–7. Although C-20 maintained a high pH of 9–10 throughout the whole observation period, MH measurements showed that its protective effect on enamel was exhausted after 20 days. This finding suggests that an apparently long-lasting alkalizing capability of C-20 was not sufficient to protect enamel against demineralization. Although the pH = 9–10 was consistently measured at the end of each four-day acid addition cycle, repeatedly exposing the enamel to the low-pH solution undersaturated with calcium and phosphate ions at the beginning of each cycle produced cumulative enamel damage, which was not remineralized over the rest of the cycle despite the alkaline pH being attained towards the end of each cycle. This reasoning also implies that the commonly used approach of measuring the alkalizing effect of restorative materials after particular time intervals^2, 4, 6, 38^ does not necessarily indicate protection against demineralization because it does not take into account the demineralization that occurs before reaching a particular time point.

Whereas all investigated commercial restorative materials showed a better protective effect than the control composite, the best performance among them was identified in a recently launched “alkasite” restorative material (Cention). In addition to showing a transient alkalizing capability, this material also showed the longest protective effect among the commercial materials. It is interesting to note that the other two commercial materials protected enamel better than the control composite, even though their pH curves indicated an acid neutralization capability similar to that of the control. Their protective effect was apparently more reliant on ion release, particularly of fluoride ions which can be incorporated into demineralized enamel over a 4-day acid exposure cycle, thereby rendering enamel more acid-resistant to the acid attack in the next cycle^39^.

The results for the BG-functionalized composites show that (I) the protective and alkalizing effects were dose-dependent, and (II) the protective and alkalizing effects were better for composites functionalized with the conventional BG 45S5 compared to the experimental fluoride-containing BG. Since these differences imply various extents of material degradation due to the dissolution of functional fillers^11^, the ion release profiles vs. the stability of mechanical properties of experimental composites with these two BG types are being addressed in ongoing studies. These studies will also include the commercial material Cention since the evidence of its high reactivity (protective and alkalizing effects better than all other tested materials except C-20) makes it an interesting candidate for studying long-term degradation of mechanical properties.

Although Cention lacks true self-adhesive properties, it was initially marketed as capable of being placed without an adhesive system due to the assumed capability for sealing marginal gaps via hydroxyapatite precipitation. These recommendations have recently been revised by the manufacturer, indicating that an adhesive system (preferably the proprietary product Cention Primer) should be applied before restoring the cavity using Cention. Therefore, clinical application of this new “alkasite” material type can be regarded as being similar to other dual-curing bulk-fill composites with the additional benefit of protective effect on surrounding dental hard tissues through ion release and alkalization.

The SEM micrographs support the finding of the protective effect of restorative materials on enamel. The anti-demineralizing effect was identified as the grinding scratch pattern on the enamel surface remaining visible over a material-dependent period of acid immersion^30^. As the protective effect was exhausted by the periodical replenishing of the acid solution, the scratch pattern progressively became shallower and was eventually replaced by etching patterns. An illustrative example of such a gradual transition was observed for C-20, which showed a gradual thinning of the scratch pattern over 4–16 days, followed by an emergence of the etching pattern after 32 days.

Distinctive etching patterns of Type 1 and 2 according to Silverstone et al.^40^ were observed on SEM micrographs. For example, Type 1 etching pattern characterized by enamel dissolution being more pronounced in prism cores was observed after 32 days for the control group, while Type 2 etching pattern in which dissolution occurs preferentially on prism peripheries was observed after 32 days for C-20 and ChemFil. Another notable finding is a layer of mineral precipitate identified after 4 days on enamel blocks immersed with Cention. Although no detailed characterization of this precipitate was performed, it was likely a hydroxyapatite layer that formed under the conditions of alkaline pH at the time point of 4 days^25^. As the pH dropped over subsequent time points, the precipitate was dissolved, revealing the underlying scratch pattern on the enamel surface.

A simple demineralization model using periodically replenished acid solution was employed in this study because it was a convenient and cost-effective means for demonstrating the protective effects of experimental composites^30^. As such a model does not simulate clinically realistic slowly-advancing subsurface lesions, a multispecies biofilm model^41^ is planned to be used in a future study that will evaluate the capability of experimental bioactive glass-containing composites for preventing the progression of carious lesions along restoration margins.

## Conclusions

Restorative materials functionalized with reactive fillers showed an anti-demineralizing effect on enamel blocks that were 5 mm away from material specimens. The remotely-acting protective effect was identified in experimental composites functionalized with two types of bioactive glass, as well as in three commercial restorative materials. Although the materials which showed pronounced alkalizing capability also showed a longer-lasting protective effect, the protection of enamel against demineralization was also attainable without a pronounced alkalization.

## Data Availability

The datasets generated during and/or analysed during the current study are available from the corresponding author on reasonable request.
